# What's With All the Hype? Applying Large Language Models in Neurology Education

**DOI:** 10.1212/NE9.0000000000200267

**Published:** 2025-11-19

**Authors:** Renzo Figari Jordan, Andrew M. Southerland

**Affiliations:** 1 Departments of Neurology and Public Health Sciences, University of Virginia, Charlottesville; and; 2 Department of Neurology, University of Virginia, Charlottesville.

The earliest publication on artificial intelligence (AI) in medical education dates back to 1992, with publications increasing rapidly starting in 2018.^1^ OpenAI's ChatGPT was released to the public in November 2022 and has since been used in a variety of contexts, including professional, academic, personal, and creative tasks. The Gartner Hype Cycle provides a graphic representation of the maturity, adoption, and social application of specific technologies (Figure). The cycle has 5 phases: innovation trigger, peak of inflated expectations, trough of disillusionment, slope of enlightenment, and plateau of productivity. At a societal level, generative AI may have entered the third phase, the trough of disillusionment.^2^ However, considering that the adoption of technology in medical education tends to lag social trends, generative AI within this domain may still be crossing the peak of inflated expectations.

**Figure:**
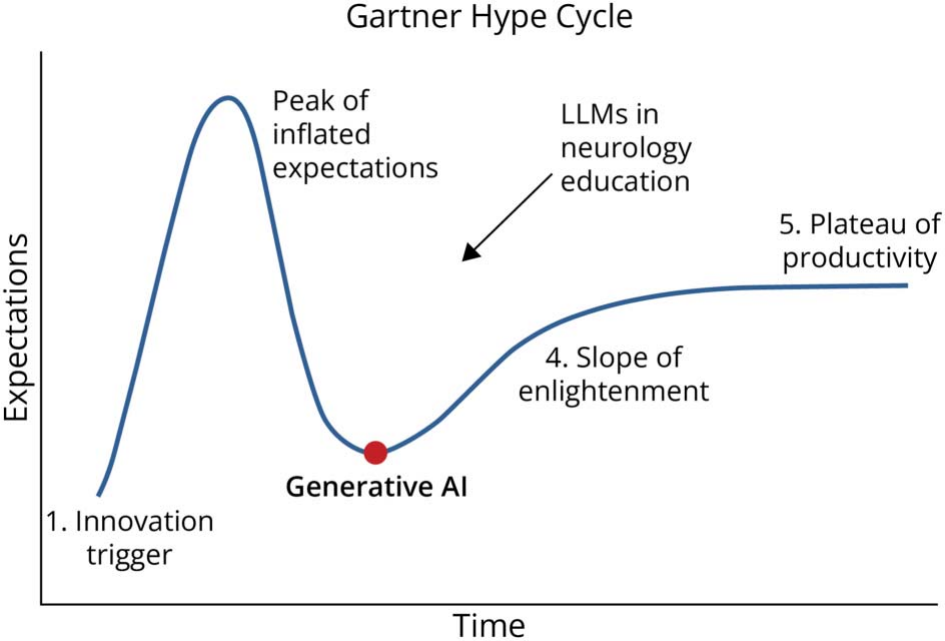
The Gartner Hype Cycle The trajectory of hype surrounding the application of AI and LLMs in neurology education. LLM = large language model.

These expectations are buttressed by numerous reports of large language models (LLMs) outperforming humans in traditional benchmarks of medical knowledge. ChatGPT-4 was the first AI model surpassing human performance on United States Medical Licensing Examination Step 1 and Step 2 questions, demonstrating 25% greater accuracy than previous models.^3^ Similarly, as it relates to neurology education, GPT-4 outperformed neurologists taking Neurology Board–style questions and the 2022 neurology specialist examination in Spain.^4,5^ Despite lacking neurology-specific training, these models achieved results equivalent to passing scores on specialized neurology examinations. When applied to clinical diagnostic reasoning vignettes, the use of GPT-4 did not enhance the performance of attending or resident physicians compared with conventional resources but did perform well in isolation, highlighting the importance of prompt formation in optimizing clinically meaningful outputs from these models.^6^

In this issue of *Neurology*® *Education*, Inojosa et al.^7^ evaluated performance on a battery of multiple-choice and open-ended questions from a postgraduate multiple sclerosis (MS) curriculum in Germany, including postgraduate trainees and students and 3 LLMs: a general-purpose large language model (GPT-4), a retrieval-augmented generation (RAG) framework (MS-RAG), and a domain-specific model trained on curated medical literature (Prof. Valmed). GPT-4 and MS-RAG achieved accuracy comparable to that of students while Prof. Valmed outperformed all groups, with nearly 10 percentage points higher accuracy (Prof. Valmed: 91.3%; GPT-4: 82.2%; MS-RAG: 86.8%; students: 82.2%). Differences emerged by question type, with LLMs performing better on single-correct multiple-choice questions but showing decreased accuracy on single-wrong questions. However, these differences did not reach statistical significance. The findings highlight that LLMs excel at pattern recognition and generating responses based on probable word sequences, whereas humans were able to reason through questions that required systematic elimination of incorrect options and avoidance of near-correct distractors. The results also corroborated a now recognizable feature of similar comparative studies: that the gap between LLM and human performance widens as questions become harder, with models outperforming students on more difficult content.

On the subset of open-ended questions, the domain-specific model Prof. Valmed outperformed other LLMs by providing no incorrect responses, but it resorted to generic nonresponses on a number of questions, highlighting the importance of balancing substantive outputs with protective guardrails when relying on LLMs for outputs.

The study has some important limitations, including the relatively small number of questions and the absence of a validated MS-specific benchmark. In addition, the level of knowledge and experience of the postgraduate MS students in the study may not fully mirror that of neurology learners in other training programs. Furthermore, the lack of student comparison for open-ended questions limited insights into differences between human and AI reasoning when it comes to free-form information retrieval.

A key takeaway from the work by Inojosa et al. is the importance of the quality of training data used to develop AI models, which greatly influences their applicability in neurology at both basic and advanced levels. Highly specialized tools such as Prof. Valmed may be effectively deployed as AI tutors capable of providing individualized feedback to students navigating complex scenarios. As models evolve with continuously updated information, they could also support formative assessments of trainees. Ready access to these tools could enhance accountability and accessibility in neurology training. Programs with limited case volumes may benefit from engaging AI in case discussions. Such applications could be adapted to support undergraduate, graduate, and subspecialty neurology education. However, simply showing that LLMs can outperform humans on knowledge and reasoning tasks is not enough; the application of these tools to augment and enhance neurology education and clinical skills needs to be researched in a formal way.

As the use of AI expands, it is our responsibility as educators to look beyond the flood of AI-related content to identify and highlight the innovations that will propel neurology into the slope of enlightenment. Teaching remains a human activity at its core, but generative AI offers myriad opportunities to enhance the human learning process, especially in cognitively rich fields such as neurology. Going forward, we must prepare ourselves and neurology learners for a pathway toward the plateau of productivity.
